# Adverse effects of long-time exposure to formaldehyde vapour on testicular tissue and sperm parameters in rats

**Published:** 2013

**Authors:** Mazdak Razi, Hassan Malekinejad, Reza Sayrafi, Mohammad Reza Hosseinchi, Sajad Feyzi, Seyed Mehdi Moshtagion, Hamed Janbaz

**Affiliations:** 1 *Department of Basic Sciences, Faculty of Veterinary Medicine, Urmia University, Urmia, Iran; *; 2 * Department of Pharmacology and Toxicology, Faculty of Veterinary Medicine, Urmia University, Urmia, Iran; *; 3 *Department of Basic Sciences, Faculty of Veterinary Medicine, Amol University of Special Modern Technologies, Amol, Iran; *; 4 *Department of Anatomy, Faculty of Veterinary Medicine, Islamic Azad University, Urmia Branch, Urmia, Iran.*

**Keywords:** Formaldehyde, Oxidative stress, Sperm, Spermatogenesis, Testosterone

## Abstract

Formalin is widely used in industry and in medicine (as tissue fixative and disinfectant).It contains reactive molecules which have been known for its cytotoxic effects. To evaluate the effect of formalin exposure on the testicular tissue and sperm parameter from neonatal period through physical and sexual maturity, 28 male Wister rats were assigned into two equal test and control groups. The test group was exposed to 1.5 ppm of the vapor of 10% formaldehyde in a special chamber for 2 hr per day at 20-26 ˚C and the air pressure of 760-763 atm. After 55 days, the tubular differentiation (TDI) and repopulation (RI) indexes in testicular tissue, sperm quality parameters, serum total antioxidant capacity and testosterone level were determined. The formaldehyde-exposed animals showed severe seminiferous tubules atrophy, edematous connective tissue, arrested spermatogenesis with negative TDI and RI and vascular thrombosis compared to control group. Histomorphological studies showed a high sperm mortality and abnormality associated with a remarkable decrease in sperm count. Formaldehyde-exposed animals revealed with decreased serum level of testosterone (*p* < 0.05) and down-regulated antioxidant status versus control group. In conclusion, the current data provide inclusive histological and biochemical information about the chronic exposure to formaldehyde with emphasizing on reproductive disorders including histological adverse effects on the testicular tissue, spermatogenesis, sperm viability, count and the abnormalities which can potentially cause infertility after sexual maturation.

## Introduction

Formaldehyde (FA, CH_2_O) is flammable, colorless, and polymerized at ambient temperature and pressure, with a pungent odour.^1^ It has been commercially produced since the early 1900s and its widespread use in a variety of applications has resulted to its hazardous exposure to people. Formaldehyde is soluble in water, ethanol and diethyl ether. It is used as polymerized form as para-formaldehyde in different fields.^2^ Air samples from schools and houses showed measurable levels of FA.^3^^,^^4^ Daily intake of FA for people who smoke at home has been estimated to be 30 to 67 µg biased on indoor air concentration of FA.^5^ A study of indoor, outdoor and personal air samples (collected from individual’s breathing zone) in Mexico City indicated that 100% of indoor and personal air samples were positive for the FA presence.^6^

Formaldehyde is an irritant compound, which can elicit adverse respiratory responses in children and adults.^7^^-^^9^ After acute inhalation, irritation of eyes, nose and throat are observed in different patients. Exposure to high concentration (>120 mg mm^-3^) of FA vapour caused hypersalivation, acute dyspnea, vomiting, vascular spasm, convulsion and finally death. Histopathological examination revealed respiratory tract disorders, bronchoalveolar constriction and lung edema.^2^^,^^10^ Previous studies have also reported FA’s adverse effects on fertility.^11^^,^^12^ Reportedly, administration of high dose of FA resulted in pathological changes in the seminiferous tubule’s (STs) of adult rats.^13^ Thus, the present study was performed on rats (between 10 and 65 days old) as a laboratory models in order to simulate condition of people exposure to FA from childhood through maturation. The histological and morphometric analyses for testicular tissue and the light microscopic analyses for sperm parameters were performed. Moreover, total antioxidant capacity (TAC) and serum level of testosterone were evaluated. 

## Materials and Methods

Twenty-eight male Wistar rats of 10 days old were used. The rats were randomly divided in two groups of test (n = 14) and control-sham (n = 14). All animals were fed with a similar standard diet and water *ad libitum* and were kept in an environmentally controlled room (20-23 ˚C and relative humidity of 50-70%, and 12hr light/12hr dark).

**Formaldehyde exposure** **. **The animals in the test group were exposed to the vapour of 10% FA in a special chamber (Adaco Co., Urmia, Iran), with the mean concentration of 1.5 ppm for 55 days. Temperature of the chamber was 20-26 ˚C and the air pressure was760-763 atm. In the control-sham group the animals were placed in the exposure chamber without any FA vapour. In non-exposure intervals the animals were kept in the laboratory animal quarters, where were far from the exposure rooms.

**Testicular weight determination. **After 55 days, the rats were euthanized (Sodium pentobarbital, 200 mg kg^-1^, IP) and the left testes were dissected out. All specimens were dissected free from surrounding tissues and weighed on a Mettler Basbal scale (Delta Range, Tokyo, Japan).

**Epididymal sperm content, quantitative sperm mortality and morphology. **The left side epididymal tissues were separated carefully from the testicle under 10 × magnification provided by stereo zoom microscope (Model TL_2_, Olympus Co., Tokyo, Japan). The epididymis tissue was divided into three segment; head, body and tail. The epididymal tail was trimmed and minced in 5 mL Ham’s F10 medium (Sigma Co., St. Louis, Mo, USA) for 20 min, 6% CO_2_, 37.5 ˚C in a CO_2_ culture device (LEEC Co., London, UK). After 20 min, epididymis was removed from the medium.^14^ The proportion of dead sperms was determined by counting 100 squares in randomly selected field from 20 smeared slides for each case, stained with Eosin-Nigrosin. In order to evaluate the sperms viability, the sperms with stained cytoplasm were considered as dead ones. The percentage of sperms with progressive motility was also evaluated. 

**Histological analyses. **On day 55, the left side testes were dissected out and fixed in Bouin’s fixative solution. Specimens were processed to paraffin embedding, cut with rotary microtome (5-6 µm) semi-serially. The special staining of Iron-Weigert’s was performed in order to clarify the germinal cells’ nucleus for tubular differentiation, repopulation indices and for tunica alboginea morphometric analyses as well. All of the specimens were studied by multiple magnifications (400 × and 1000 ×).^15^ For the quantification of cells and their dimensions in 100 seminiferous tubules (in one specimen) the 100 µm morphometric lens-device was used. The dimensions were expressed in 1 µm. The Leydig and Sertoli cells number counted per mm^2^ of the interstitial connective tissue and per one ST of 100 ST of each sample, respectively. The interstitial connective tissue vessels diameter was estimated in 200 sections (6 µm) using morphometric lens. 

**Tubular differentiation index (TDI) determination. **To estimate the TDI, the percentage of STs that were showing more than three layers of differentiated germinal cells from spermatogonia type A, 200 sections (6 µm) were prepared and the STs which showed more than three layers considered as TDI positive. 

**Repopulation index (RI) calculation. **To determine the RI, the special staining of Iron-Weigert’s was performed in order to detect the spermatogonia cells. The cells with dark stained nucleus were considered as spermatogonia type A (inactive cells) and the cells with light stained nucleus were marked as spermatogonia type B (active cells). For this purpose, the ratio of active spermatogonia to inactive spermatogonia in STs was calculated in 200 prepared sections as mentioned earlier.^14^ The percentage of tubules with positive RI analyzed and the results presented as the percentage of tubules with positive RI.

**Testosterone assessment. **Blood samples from corresponding animals were collected and serum samples were prepared by centrifugation (3000* g *for 5 min), and subjected to assessment of the serum concentration of testosterone. Testosterone was assessed by using competitive chemiluminescent immunoassay kit (Diagnosis Related Groups Co., Germany). 

**Serum TAC. **To determine the effect of FA-exposure on oxidative stress system, TAC in the control-sham and test groups were measured. The assessment was carried out based on ferric reduction antioxidant power (FRAP) assay.^15^ Briefly, at low pH which was provided using acetate buffer (300 mM, pH 3.6), reduction of Fe^III^-TPTZ complex to the ferrous form produces an intensive blue color that could be measured at 593 nm. The intensity of the complex following adding the appropriate volume of the serum to reducible solution of Fe^III^-TPTZ is directly related to total reducing power of the electron donating antioxidant. Aqueous solution of Fe^II^ (FeSO_4_.7H_2_O) and appropriate concentration of freshly prepared ascorbic acid were used as blank and standard solutions, respectively.

**Statistical analyses. **All data were analyzed using paired *t*-test to compare quantitative parameters referring to paired organs within a group. All values were expressed as mean ± SD. To determine the regression between the Leydig and Sertoli cells number the chi- square test was used. A probability of *p* < 0.05 was considered to be statistically significant. 

## Results

**General findings.** Formaldehyde inhalation had no effect on food and water consumption in the early stages of the experiment, while time-dependently the animals in test group showed a decreased food and water consumption. Formaldehyde inhalation for 55 days resulted in reduction of testicular size and weight in the test group compared to control rats. Additionally all rats in the test group showed body weight loss and fatigued appearance (Table 1). Moreover, the rats in the test group demonstrated nasal discharges and irritated eyes.

**Fig. 1:**
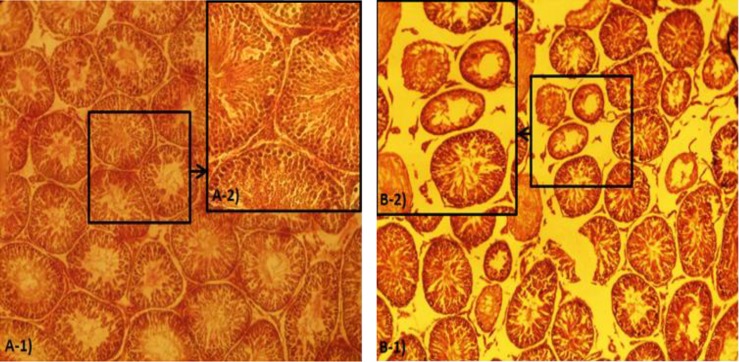
Cross section of the rat testes; (**A-1 and A-2**) control group, normal seminiferous tubules with no edema in interstitial connective tissue, (**B-1 and B-2**) The FA-exposed group, severe interstitial edema. Note the seminiferous tubules depletion in B-2, (Iron-Weigert’s staining, A-1 and B-1:100×, A-2 and B-2: 400×).

**Fig. 2:**
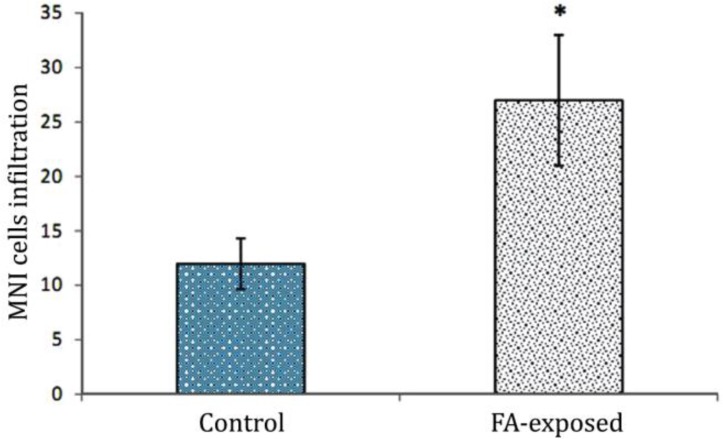
Effect of FA exposure on mononuclear immune (MNI) cells infiltration in connective tissue. * Asterisk indicates significant differences between the control and FA-exposed groups (*p* < 0.01), (n = 14).

**Histological alteration.** Histological examinations revealed that in the FA-exposed animals the thickness of tunica albuginea was increased. Sub-capsular and perivascular edema was demonstrated in the test group. A remarkable thrombosis in the capsular and interstitial tissue vessels was demonstrated. The mononuclear immune cells infiltration was exhibited in connective tissue of the FA-exposed group (Fig. 1). Meanwhile, no histological changes were observed in the control group. The data for immune cell infiltration is presented in Fig. 2. 

The severe degeneration in germinal epithelium cells was revealed in more than 50% of the STs in the FA-exposed animals. In addition spermatozoids were rarely manifested in the lumen of the ST’s and the spermatogenesis process seemed arrested (Fig. 1). Observations demonstrated that the animals in the FA-exposed group showed remarkable increase in percentage of STs with germinal epithelium dissociation. The germinal epithelium was reduced in height in the test group. 

**Table 1:** Effect of FA exposure on testicular weight, length, width and total body weight. Data are presented as mean ± SD

**Parameters**	** Control**	** FA-exposed**
**Testicular weight (g)**	0.89 ± 0.10	0.746 ± 0.11*
**Testicular length (mm)**	19.87 ± 1.25	15.99 ± 0.83*
**Testicular width (mm)**	12.85 ± 0.69	9.66 ± 1.00*
**Total body weight (g)**	247.38 ± 4.77	196.57 ± 8.90*

* Asterisk indicate significant differences between the control and FA-exposed groups (*p* <0.05).

**Fig. 3:**
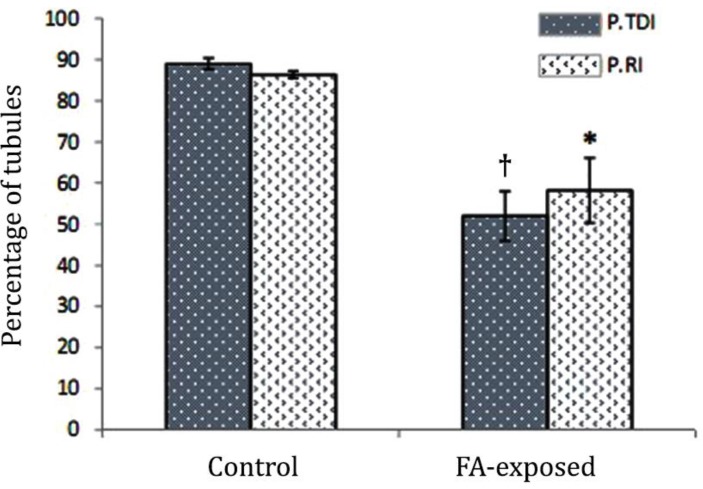
Effect of FA exposure on percentage of tubules with positive tubular differentiation (P.TDI) and repopulation (P.RI) indices. Symbols present significant differences between the control and FA-exposed groups (*p* < 0.05), (n = 14).

Accordingly, the animals in the FA-exposed group showed a remarkable increase in STs with negative TDI. The histological analyses for RI showed an increased percentage of tubules with negative RI in the FA-induced rats (Fig. 3). The histomorphometric data for testicular tissue are presented in Table 2. 

**Fig. 4:**
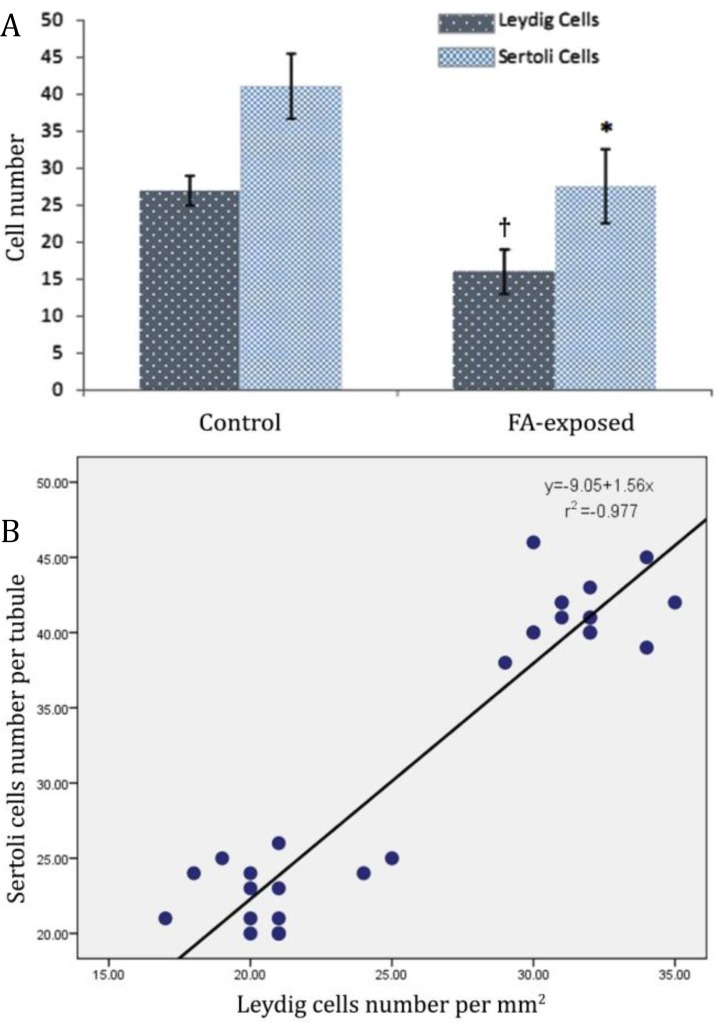
Effect of FA exposure on **A) **Leydig cells distribution in one mm^2^ of interstitial connective tissue and Sertoli cells per each seminiferous tubule; Symbols present significant differences between the control and FA-exposed groups (p < 0.05), (n = 14). **B)** correlation between decreased Leydig cells distribution (per mm^2^ of interstitial tissue) with reduced number of Sertoli cells in one seminiferous tubule. There is negative correlation (r^2 ^= -0.97).

**Fig. 5:**
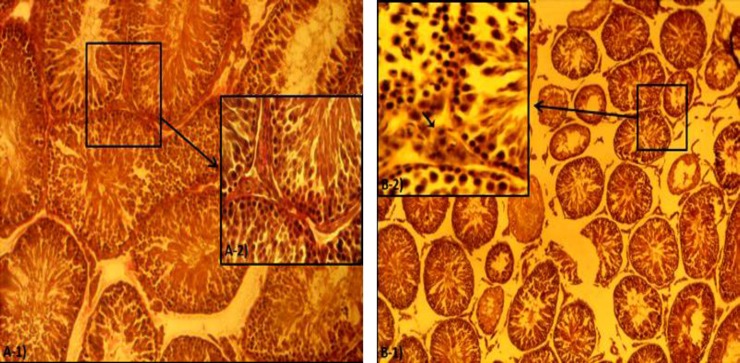
Cross section from testes; **(A-1 and A-2)** control group, Leydig cells are presented normal. **(B-1 and B-2)** The hyper-trophied Leydig cells in the FA-exposed rats (note Fig. B-2), (Iron-Weigert’s staining, A-1 and B-1: 100×, A-2 and B-2: 400×).

Histological analyses demonstrated an obvious decrease in Leydig cells number per mm^2^ of interstitial connective tissue associated with cellular hypertrophy. The Sertoli cells number per each seminiferous tubule decreased in the FA-induced animals in comparison to the control group (Figs. 4 and 5). 

**Sperm parameters. **The animals in FA-exposed group showed a significant (*p* < 0.05) decrease in sperm count compared to the control group. The especial staining for sperm viability showed that FA-exposure significantly (*p* < 0.05) increased sperm mortality in the test animals. 

**Table 2:** Histomorphometric analyses of testicular tissue in control and FA-exposed animals. Data are presented as mean ± SD.

**Parameters**	**Control**	**FA-exposed**
**Germinal epithelium height (µm) **101.28 ± 4.21		51.03 ± 3.75*
**Tunica albuginea thickness (µm)**	78.72 ± 5.24	98.34 ± 6.56*
**Vascular diameter (µm)**	66.50 ± 5.28	96.82 ± 2.70*

* Asterisk indicate significant difference between the control and FA-exposed groups (*p* < 0.05).

Formaldehyde inhalation resulted in a significant increase in sperm abnormality (*p* < 0.05), accordingly the animals in the FA-exposed group revealed up to 75% morphologically immature sperms (sperms with cytoplasmic droplet). The percentage of motile sperms significantly (*p* < 0.05) reduced in the FA-induced rats. The data for sperm parameters are presented in Table 3.

**Fig. 6:**
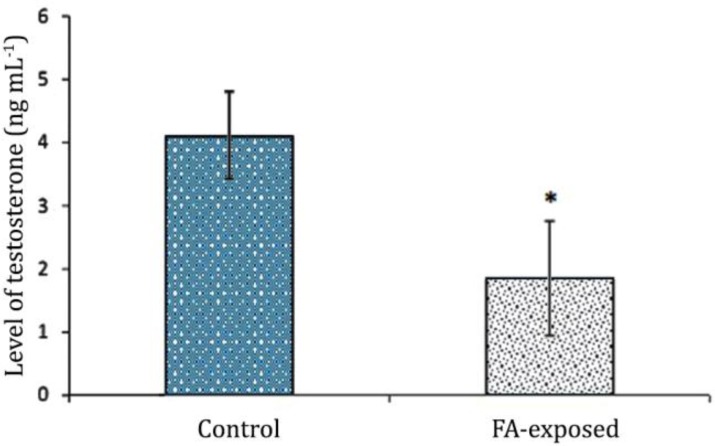
Effect of FA exposure on serum levels of testosterone. Asterisk presents a remarkable difference between the control and FA-induced groups (*p* < 0.05), (n = 14).

**Fig. 7:**
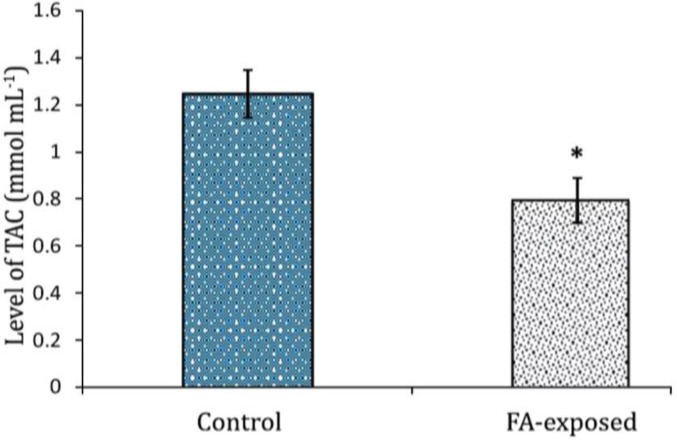
Effect of FA exposure on serum total antioxidant capacity. Asterisk indicates significant difference between the control and FA-exposed groups (*p* < 0.05), (n = 14).

**Serum level of testosterone and TAC.** Hematological observations demonstrated that the serum level of testosterone significantly decreased in the FA- exposed group, while the animals in the control group showed normal level of testosterone (*p* < 0.05), (Fig. 6). The animals in FA-exposed group manifested with significantly (p < 0.05) decreased level of TAC in comparison to control group. The data for serum TAC are presented in Fig. 7.

**Table 3:** Effect of formaldehyde exposure on sperm count, motility, viability and abnormality (cytoplasmic droplet) in the control and FA-exposed groups. Data are presented as mean ± SD.

**Parameters**	**Control**	**FA-exposed**
**Count (×10** ^6^ **)**	56.50 ± 3.10	27.75 ± 4.03*
**Motility (%)**	88.00 ± 1.82	35.57 ± 4.43*
**Viability (%) **	89.50 ± 1.29	40.75 ± 1.26*
**Abnormality (%)**	9.85 ± 1.66	76.27 ± 4.53*

* Asterisks indicate remarkable differences (*p* < 0.05) between the control and FA-exposed animals.

## Discussion

Formaldehyde is a common environmental contaminant. Despite enormous efforts to reduce the formaldehyde-related hazards, exposure to FA remains one of the most crucial occupational and environmental health problem.^17^ Occupational exposure to FA may occur during manufacturing, processing and during the use of FA containing products, mainly via the dermal and inhalation routes. 

According to previous studies, intraperitoneal administration of FA at dose levels of 0.2, 2 and 20 mg kg^-1^ could cause degeneration and necrosis of spermatozoid in adulthood rats.^13^ Cowdhury *et al*. found low Leydig cells distribution with spermatogenesis arrest in 5, 10 and 15 mg kg^-1^ intraperitoneally FA-injected rats.^18^^-^^20^ Formaldehyde vapour can increase HSP_70_ synthesis in STs in the rats.^21^ Microscopic analyses showed that the spermatogenesis significantly inhibited by FA.^22^ In corroboration with previous reports, in this study, histological investigations revealed arrested spermatogenesis with atrophied STs in the test groups. Additionally observations demonstrated that the high proportion of spermatogenic cells was dissociated from basal membrane of STs and were observed with piknotic nuclei. Giant cells were demonstrated in the FA-exposed rats STs, locating close to basement membrane, suggesting karyokinesis in the FA-induced rats. According to Sarsilmaz *et al*., exposure to FA (10 and 20 ppm) caused considerable decrease in Leydig cells of mouse testis.^11^ In the present study animals in the test group showed decreased Leydig cells with cytoplasmic granulation, indicating that early exposure to FA (even in neonates) can result in severe reduction of Leydig cells. At the same time serum level of testosterone significantly reduced in the FA-exposed rats which may indicate that the chronic exposure to FA (from early ages until maturation age) results in Leydig cells malfunction. Moreover Leydig cells are the main cells in testicular tissue which control Sertoli cells normal and physiological function. Thus, any disruption between Leydig and Sertoli cells can influence spermatogenesis process and ultimately blocks spermatocytogenesis and spermatogenesis processes. Our histological analyses for TDI and RI indexes confirmed this hypothesis. The histopathological changes in spermatogenesis process and the STs have been explained by different mechanisms including the cytotoxic effect of FA and inhibition of nucleic acid and proteins synthesis in spermatogenic cells.^23^^,^^24^

Formaldehyde can increase the production of reactive oxygen species in most of the target tissues.^25^^,^^26^ Formaldehyde inhalation decreases the effectiveness of the testicular antioxidant system.^21^ Our antioxidant analyses for serum level of TAC showed a significant reduction of TAC in the FA-exposed animals. Since the sperm plasma membrane consists of a high content of polyunsaturated fatty acids, therefore is highly vulnerable to damage by free radicals.^14^^,^^27^ The special staining (Eosin-Nigrosin staining) showed a high percentage of dead sperms with stained cytoplasm in the FA-induced group, which might be related to the free radical-induced lipid peroxidation and consequently might reduce the spermatozoa motility.^28^^,^^29^ Our analyses for sperm motility showed that long-term exposure to FA decreased sperm motility. This finding suggests that FA affects the sperm motility from very early ages and the impairment develops eventually on maturation age of the rats. 

There is a positive correlation between abnormal and immature sperm velocity with oxidative stress.^30^ Our biochemical and histological observations supported this correlation as the FA-exposed animals with increased percentage of immature, immotile and dead sperms showed the decreased level of TAC in serum. Sperm quality and quantity are critical factors to the male fertility.^31^^,^^32^ In the present study, we demonstrated that the percentage of abnormal sperms were significantly increased in the test group. A similar finding has been already reported by Ozen *et al*.^33^ Thus, it might be concluded that the chronic inhalation of FA can increase abnormal sperms. Therefore, early exposure to FA can result in drastic reduction in semen quality which in turn will lead to remarkable fertility problems.

In conclusion, chronic inhalation of 10% formaldehyde from very young age through maturation (60-65 days-old) in rats can cause histological adverse effects on testicular tissue, spermatogenesis, and sperm viability. 
